# Supplementary material for: Nesting Ecology of European Hedgehogs (Erinaceus europaeus) in Urban Areas in Southeast Spain: Nest Habitat Use and Characteristics
Source: Animals (Basel). 2023 Jul 29;13(15):2453. doi: 10.3390/ani13152453 (PMC10417487; doi:10.3390/ani13152453)
Supplement: Supplementary file 1 [file animals-13-02453-s001.zip › animals-2503226-supplementary.pdf]

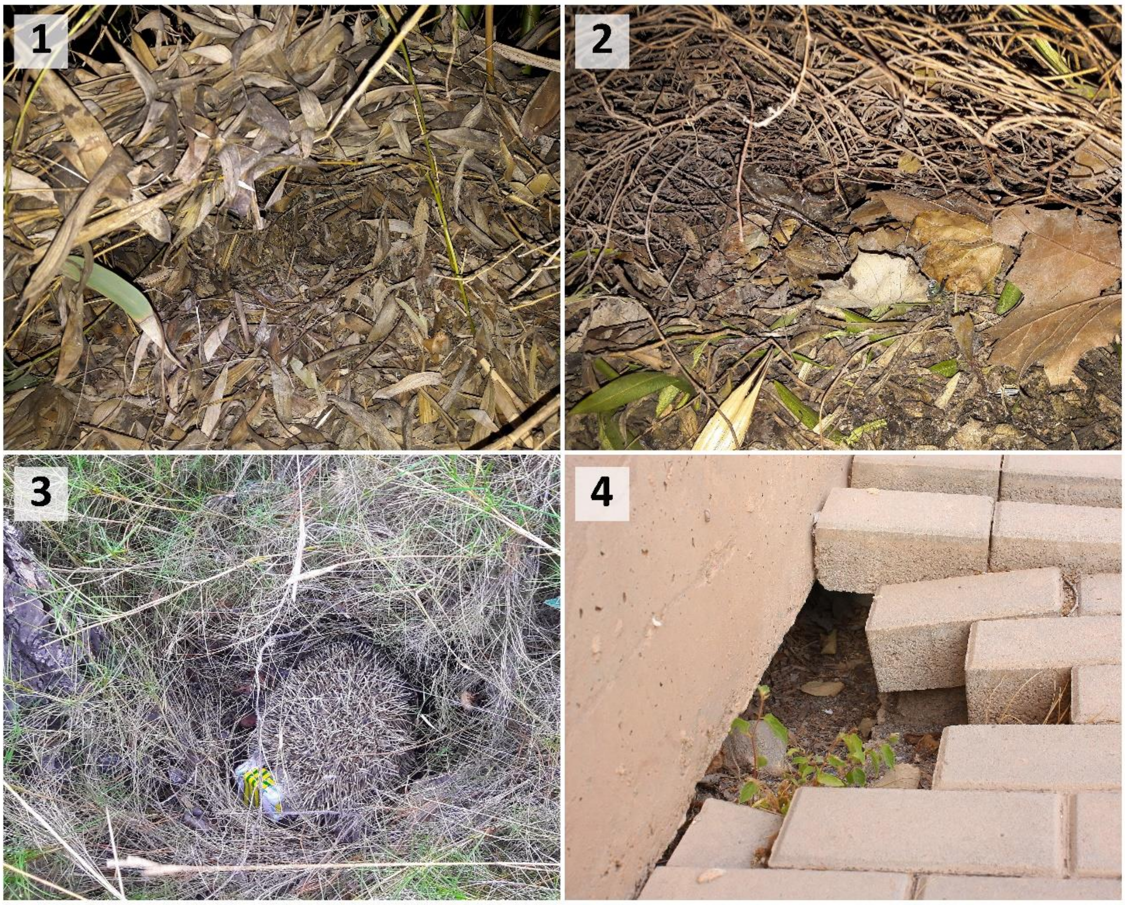


**Supplementary Figure S1.** Examples of different types of hedgehogs' nests. **1)** Well-structured nest, with a fairly amount of nesting material (bamboo leaves) accumulated around the hedgehog and covering it forming a compact dome. **2)** Poorly structured nest, with some nesting material presumably carried by the hedgehog (dry tree leaves, fresh bush leaves and some plastics) but without forming a compact structure around it. **3)** Unstructured nest with the hedgehogs simply lying on the vegetation. **4)** Burrow, taking advantage of cracks in both artificial and natural structures.

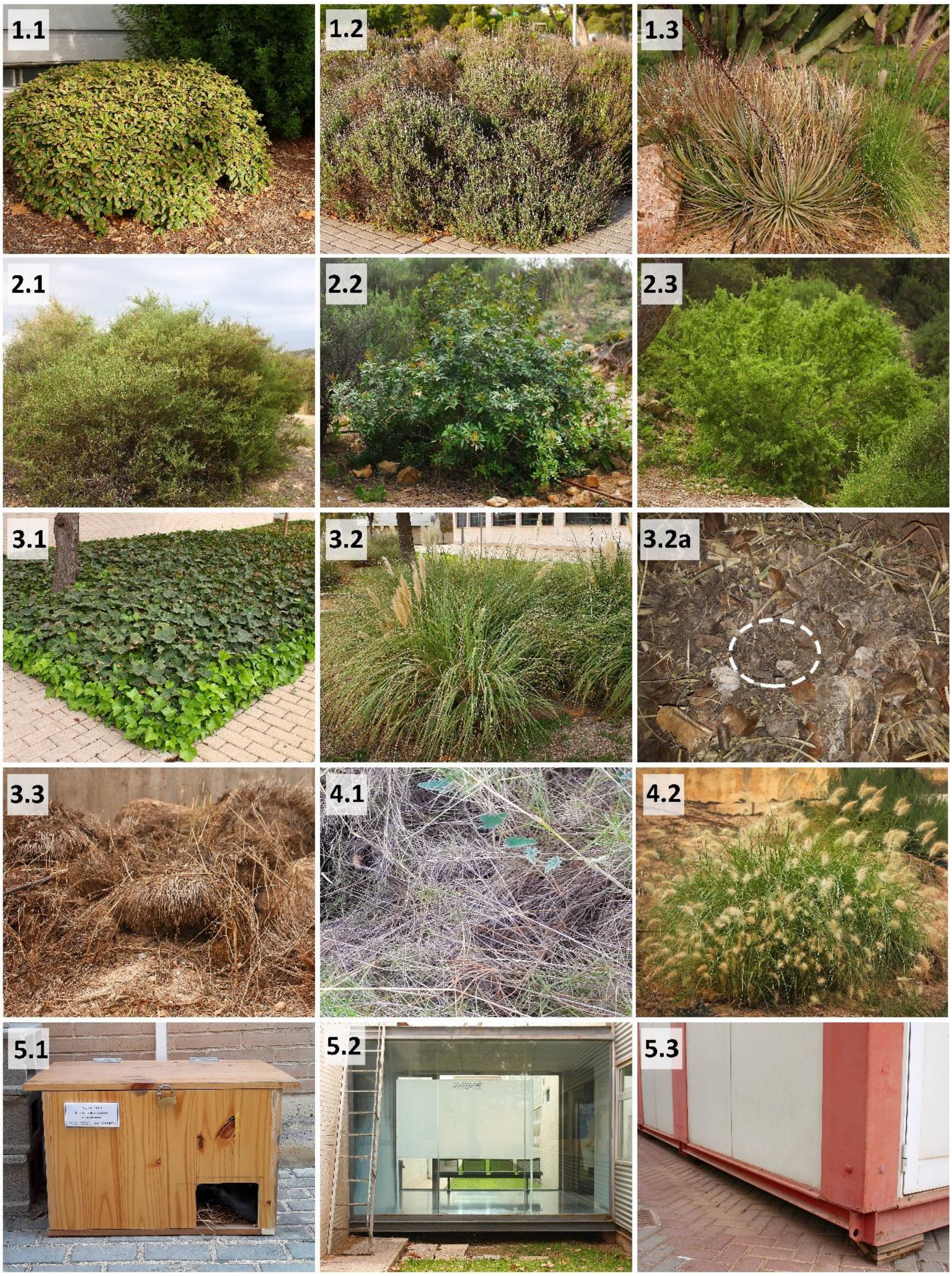


**Supplementary Figure S2.** Examples of different main hosting structures used by hedgehogs to install their nests. **1)** Bush-like plants from university campus (1.1. *Pittosporum sp.*; 1.2. *Lavandula* sp. & only used by males: 1.3 Branched cactus – *Agave sp.*); **2)** Bush-like plants from the urban forest (2.1. *Salsola oppositifolia*. & only used by males: 2.2. *Pistacea lentiscus*; 2.3 *Rhamnus lycioides*); **3)** Sturdy plants from the university campus (3.1. Ivy – *Hedera helix*; 3.2. Pampa grass – *Cortaderia selloana*; 3.2a. Detail of a pampa grass cut by the gardener, in which a hedgehog appeared inside (dotted white circle); 3.3. Death Palm tree stump); **4)** Herbaceous plants (4.1. *Brachypodium retusum*; 4.2. Exotic garden grass); **5)** Artificial structures from the university campus (5.1. Wooden box; 5.2. Building; 5.3. Office trailer).

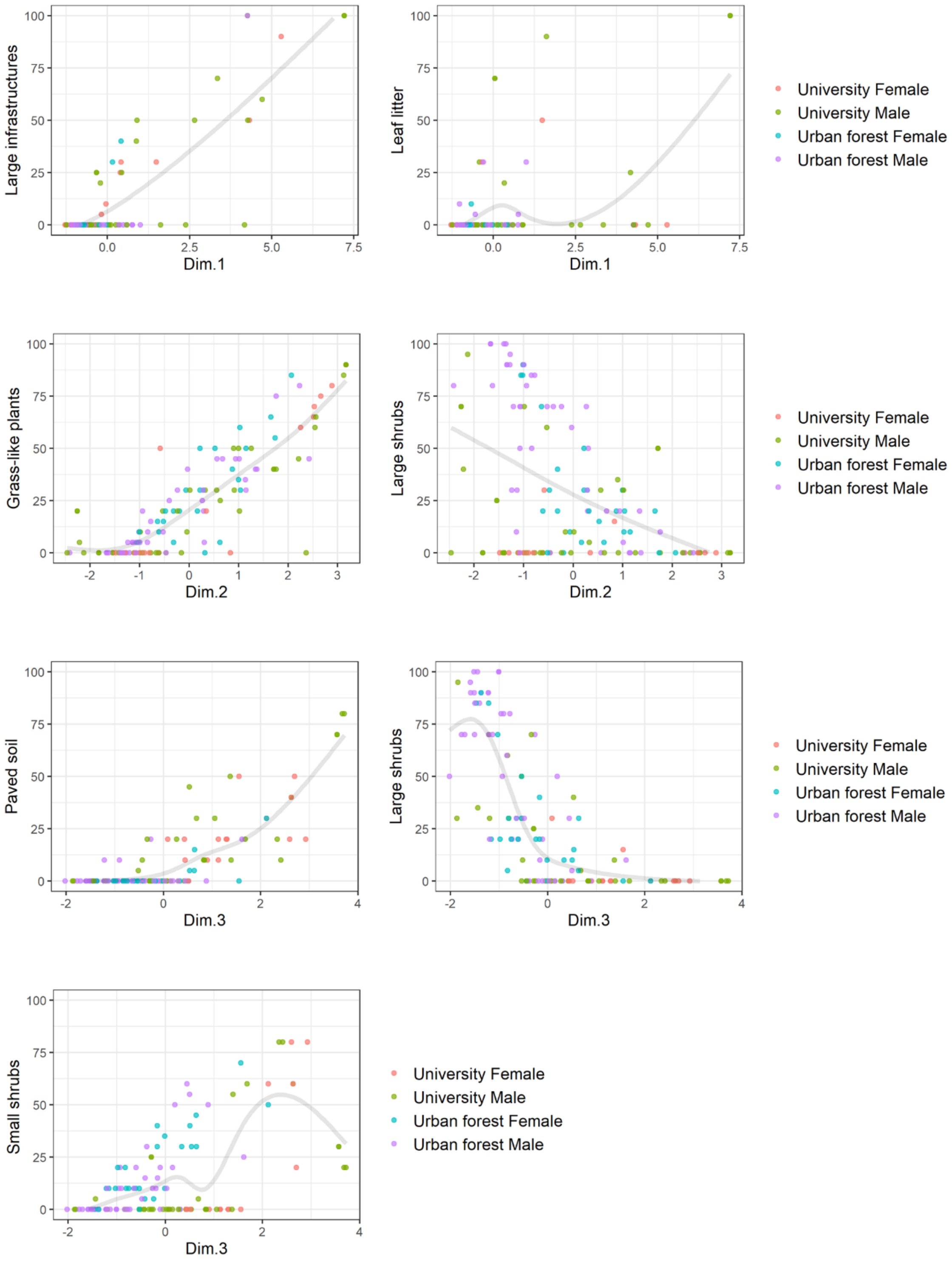
Large infrastructures
Dim.1
Leaf litter
Dim.1
University Female
University Male
Urban forest Female
Urban forest Male
Grass-like plants
Dim.2
Large shrubs
Dim.2
University Female
University Male
Urban forest Female
Urban forest Male
Paved soil
Dim.3
Large shrubs
Dim.3
University Female
University Male
Urban forest Female
Urban forest Male
Small shrubs
Dim.3
University Female
University Male
Urban forest Female
Urban forest Male

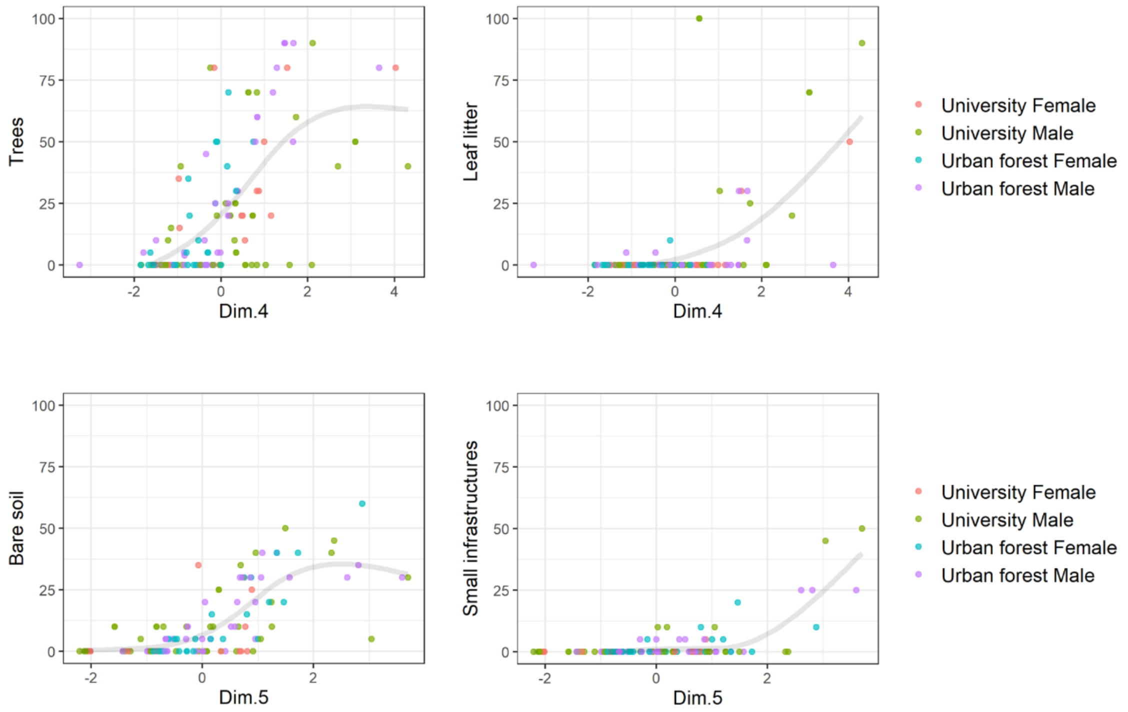


**Supplementary Figure S3.** Relation between the quantitative variables describing the habitat in a 5-meter radius around hedgehog nests and the first five dimensions resulting from FAMD. Only the variables with the strongest correlation with each dimension ($R^2 > 0.2$) are shown. Each nest is represented by a point coloured according to study area and sex. The trend is represented with a grey line calculated using a Generalized Additive Model (GAM).

**Supplementary Table S1.** Descriptive statistics (mean, standard deviation, SD, and sample size, N) for the variables describing nest use (number of nests per individual, number of times that each nest was used, distance between consecutive nests in m), their spatial spread (MCP, minimum convex polygon, ha) and distance (D. in m) to some artificial structures.

| | University campus | | | | Urban forest | |
|---|---|---|---|---|---|---|
| Season | Spring-Summer | | Winter | | Spring-Summer | |
| Sex | Female | Male | Female | Male | Female | Male |
| **No. nests** | 1.00 | 4.20 | 2.80 | 3.14 | 4.33 | 5.00 |
| SD | 0.00 | 1.30 | 0.45 | 0.90 | 1.97 | 1.80 |
| N | 2 | 5 | 5 | 7 | 6 | 9 |
| **D. consecutive nests** | 0.00 | 177.00 | 119.40 | 145.00 | 41.50 | 114.89 |
| SD | 0.00 | 84.40 | 122.77 | 136.47 | 36.30 | 95.10 |
| N | 2 | 5 | 5 | 7 | 6 | 9 |
| **MCP** | - | 7.21 | 0.71 | 1.41 | 0.42 | 2.42 |
| SD | - | 6.05 | 0.66 | 1.34 | 0.63 | 2.40 |
| N | 0 | 4 | 4 | 5 | 5 | 8 |
| **No. Times** | 6.00 | 2.19 | 1.93 | 1.91 | 2.65 | 2.16 |
| SD | 1.41 | 1.69 | 0.73 | 1.72 | 3.24 | 2.04 |
| N | 2 | 21 | 14 | 22 | 26 | 45 |
| **Distance to artificial structures** | | | | | | |
| **D. path** | 1.00 | 25.39 | 3.95 | 23.57 | 19.95 | 13.50 |
| SD | 0.00 | 35.26 | 6.49 | 40.07 | 16.97 | 8.93 |
| N | 2 | 21 | 14 | 22 | 26 | 45 |
| **D. road** | 117.83 | 52.73 | 77.69 | 58.58 | 47.44 | 101.38 |
| SD | 112.89 | 46.80 | 57.08 | 62.98 | 50.98 | 59.23 |
| N | 2 | 21 | 14 | 22 | 26 | 45 |
| **D. feeder** | 178.46 | 202.18 | 101.41 | 161.03 | 102.18 | 143.48 |
| SD | 126.52 | 187.11 | 109.12 | 174.76 | 80.74 | 78.36 |
| N | 2 | 21 | 14 | 22 | 26 | 45 |
| **D. streetlight** | 8.99 | 16.31 | 8.76 | 12.95 | 23.31 | 35.68 |
| SD | 3.09 | 13.01 | 4.07 | 5.99 | 15.30 | 24.52 |
| N | 2 | 21 | 14 | 22 | 26 | 45 |
